# The ETS transcription factor ETV5 is a target of activated ALK in neuroblastoma contributing to increased tumour aggressiveness

**DOI:** 10.1038/s41598-019-57076-5

**Published:** 2020-01-14

**Authors:** Liselot M. Mus, Irina Lambertz, Shana Claeys, Candy Kumps, Wouter Van Loocke, Christophe Van Neste, Ganesh Umapathy, Marica Vaapil, Christoph Bartenhagen, Genevieve Laureys, Olivier De Wever, Daniel Bexell, Matthias Fischer, Bengt Hallberg, Johannes Schulte, Bram De Wilde, Kaat Durinck, Geertrui Denecker, Katleen De Preter, Frank Speleman

**Affiliations:** 1 Department of Biomolecular Medicine, Ghent University, Ghent, Belgium; 2 Cancer Research Institute Ghent (CRIG), Ghent, Belgium; 3 Department of Uro-gynaecology, Ghent University Hospital, Ghent, Belgium; 4 Department of Medical Biochemistry and Cell Biology, Institute of Biomedicine, Sahlgrenska Academy, University of Gothenburg, Gothenburg, Sweden; 5 Translational Cancer Research, Lund University, Lund, Sweden; 6 Department of Experimental Pediatric Oncology, University Children’s Hospital of Cologne, Medical Faculty, University of Cologne, 50937 Cologne, Germany; 7 Centre for Molecular Medicine Cologne (CMMC), University of Cologne, 50931 Cologne, Germany; 8 Department of Paediatric Haematology and Oncology, Ghent University Hospital, Ghent, Belgium; 9 Laboratory of Experimental Cancer Research, Ghent University, Ghent, Belgium; 10 Department of Paediatric Oncology and Haematology, University Children’s Hospital Essen, Essen, Germany; 11 Department of Paediatric Oncology and Haematology, Charité University Medical Centre Berlin, Berlin, Germany; 12 German Cancer Consortium (DKTK), Berlin, Germany; 13 German Cancer Research Centre (DKFZ), Heidelberg, Germany

**Keywords:** Molecular medicine, Paediatric cancer

## Abstract

Neuroblastoma is an aggressive childhood cancer arising from sympatho-adrenergic neuronal progenitors. The low survival rates for high-risk disease point to an urgent need for novel targeted therapeutic approaches. Detailed molecular characterization of the neuroblastoma genomic landscape indicates that *ALK-*activating mutations are present in 10% of primary tumours. Together with other mutations causing RAS/MAPK pathway activation, *ALK* mutations are also enriched in relapsed cases and *ALK* activation was shown to accelerate *MYCN*-driven tumour formation through hitherto unknown *ALK*-driven target genes. To gain further insight into how ALK contributes to neuroblastoma aggressiveness, we searched for known oncogenes in our previously reported *ALK*-driven gene signature. We identified ETV5, a *bona fide* oncogene in prostate cancer, as robustly upregulated in neuroblastoma cells harbouring *ALK* mutations, and show high ETV5 levels downstream of the RAS/MAPK axis. Increased ETV5 expression significantly impacted migration, invasion and colony formation *in vitro*, and *ETV5* knockdown reduced proliferation in a murine xenograft model. We also established a gene signature associated with *ETV5* knockdown that correlates with poor patient survival. Taken together, our data highlight ETV5 as an intrinsic component of oncogenic *ALK*-driven signalling through the MAPK axis and propose that ETV5 upregulation in neuroblastoma may contribute to tumour aggressiveness.

## Introduction

Neuroblastoma is the most common extracranial solid paediatric tumour, accounting for 15% of all childhood cancer deaths and arises from the developing sympathetic nervous system^1^. In patients with high-risk disease, 5-year event-free survival is less than 50%^2^, with survivors often suffering from severe side-effects associated with current intensive multi-modal therapies and relapse after first-line therapy^1^. Recent sequencing efforts have established the mutational landscape of primary and relapsed neuroblastoma. Primary neuroblastomas, similar to other embryonic tumours, have a very low mutation burden. The ‘*anaplastic lymphoma kinase*’ (*ALK*) tyrosine kinase receptor is the only target with significant recurrent mutations occurring in up to 10% of sporadic cases and a small fraction of germline mutations^3–5^. Of further interest, *ALK* mutations, together with other mutations affecting the RAS/MAPK pathway, appear to be further enriched in relapsed cases, either through selection of minor subclones present at diagnosis or mutations arising during therapy^6^. Recently, ALKAL1 and ALKAL2 were identified as potent ligands binding to the extracellular domain of ALK^7^. In addition to neuroblastoma, ALK activation also occurs in other tumour entities, most notably through activating fusion genes in non-small cell lung cancer (NSCLC) and anaplastic large cell lymphoma (ALCL)^8^.

We previously reported that combined occurrence of *MYCN* amplification and the *ALK*^*F1174L*^ mutation in primary tumours resulted in a very aggressive tumour phenotype in patients^3^. Moreover, further *in vivo ALK*^*F1174L*^ modelling showed drastic acceleration of *MYCN*-driven tumour formation in transgenic mice and zebrafish^9,10^. One possible mechanism through which *ALK* mutations may render neuroblastoma more aggressive is through increased MYCN activity resulting from PI_3_K-directed activation of *ERK5* transcription levels or inhibiting *GSK*_3_*ß*-mediated repression of MYCN protein degradation^11,12^. Further, we recently identified a novel mutant ALK-controlled mechanism driving MYCN activity through PI_3_K/AKT-FOXO3a-controlled downregulation of HBP1, the latter being a negative regulator of MYCN^13^. IRS2 was also recently revealed as an important protein mediating survival through the PI_3_K/AKT-FOXO3 signalling axis^14^.

We previously showed that ALK signals both through the MAPK and PI_3_K/AKT pathways in neuroblastoma cells^5^. We now further explored our previously established 77-gene signature driven in neuroblastoma by constitutive ALK signalling, after ALK inhibition, and identify ETV5 as induced by the ALK mutant protein^5^. ETV5 is a member of the polyomavirus enhancer activator 3 (PEA3) subfamily of the E26 transformation specific gene (ETS) transcription factors known to be part of the MAPK signalling pathway. We further investigated ETV5 in neuroblastoma models, given its established developmental role in neural crest cell lineage decisions and neuronal progenitor cell proliferation^15^, to further explore the MAPK axis of ALK signalling. We confirm and further extend the findings by Lopez-Delisle *et al*. (2018), and functionally characterize the role of elevated ETV5 in the neuroblastoma phenotype *in vitro* and *in vivo*. We demonstrate that elevated ETV5 contributes to migration, clonogenic potential and invasive properties of human neuroblastoma cells while ETV5 knockdown in a xenograft model attenuates proliferation and tumour growth. Finally, we established a gene signature associated with *ETV5* knockdown, which correlates with poor overall survival in patients with neuroblastoma.

## Results

### ETV5 is transcriptionally regulated by activating ALK mutations or receptor-ligand stimulation

**Figure 1:**
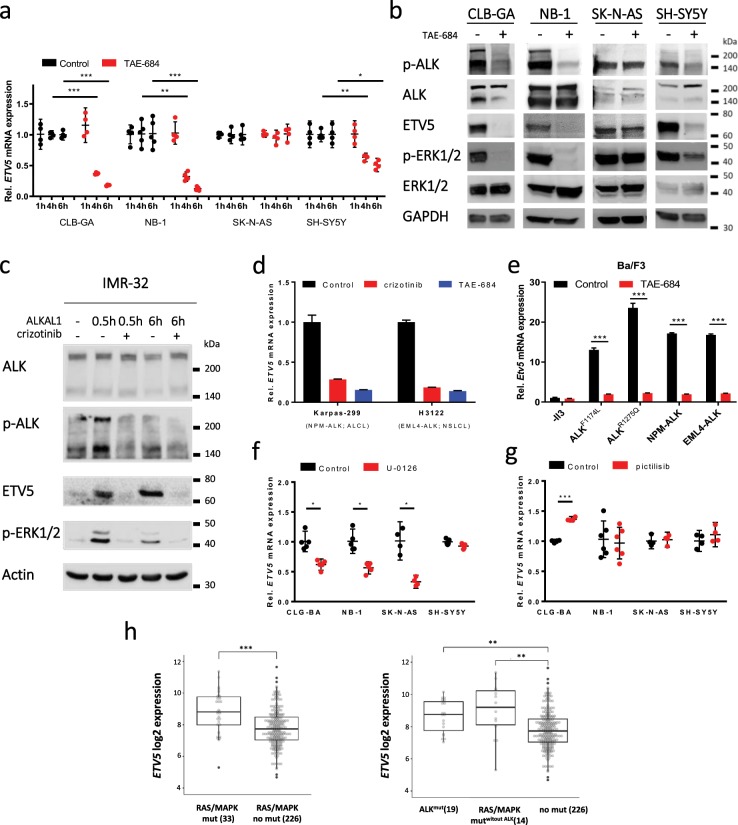
ALK regulates ETV5 expression through the MAPK signalling pathway. (**a**) Relative *ETV5* mRNA expression levels in four different neuroblastoma cell lines treated for indicated time periods with a vehicle control (DMSO) or the ALK inhibitor, TAE-684 (0.3 µM). (n_CLB-GA, SK-N-AS, SH-SY5Y_ = 4; n_NB-1_ = 5; mean with error bars representing 95% CI after error propagation with mean centring and scaling to control). (**b**) Western blot analysis for p-ALK, total ALK, ETV5, p-ERK1/2 and total ERK1/2 in four different neuroblastoma cell lines after ALK inhibition with TAE-684 (0.3 µM, 6 h). (cropped images, full-length images are presented in Supplementary Figs. S5–6). (**c**) Western blot analysis for p-ALK, total ALK, ETV5 and p-ERK1/2 in IMR-32 after stimulation with ALK ligand (ALKAL1) for 30 min or 6 h and subsequent treatment with ALK inhibitor, crizotinib (0.25 µM). (cropped images, full-length images are presented in Supplementary Fig. S7a). (**d**) Relative *ETV5* mRNA expression levels in non-neuroblastoma ALKoma tumour cell lines Karpas-299 (ALCL) and H3122 (NSCLC) after a 6 h treatment with a vehicle control (DMSO) or the ALK inhibitors, crizotinib (0.5 µM) or TAE-684 (0.3 µM). (n = 1; mean with error bars representing SD after error propagation). (**e**) Relative *Etv5* mRNA expression levels in *ALK*-dependent Ba/F3 cells 5 h post-treatment with a vehicle control (DMSO) or the ALK inhibitor, TAE-684 (0.3 µM). (n = 1; mean with error bars representing SD after error propagation). (**f**,**g**) Relative *ETV5* mRNA expression levels in four different neuroblastoma cell lines after a 6 h treatment with a vehicle control (DMSO) or the MEK inhibitor, U-0126 (8 µM) and the PI_3_K inhibitor, pictilisib (500 nM). (n_CLB-GA, NB-1, SH-SY5Y; U-0126_ = 5; n_SK-N-AS; U-0126_ = 4; n_CLB-GA, SK-N-AS, SH-SY5Y; pictilisib_ = 4; n_NB-1, pictilisib_ = 6; mean with error bars representing 95% CI after error propagation with mean centring and scaling to control). (**h**) Boxplot representation of the log2 *ETV5* mRNA expression levels in a large independent primary neuroblastoma cohort (GSE49711 dataset) with (left, 33 cases) and without RAS/MAPK pathway mutations (right, 226 cases) (GSE120572, left panel); and for cases with mutant ALK (ALK^mut^) or mutations in other RAS/MAPK pathway genes (Mut^without ALK^) (right panel). (*p < 0.05; **p < 0.01; ***p < 0.001).

In follow-up to our initial study, which established a 77-gene signature driven by constitutive ALK signalling^5^, we performed a time series analysis of *ETV5* expression levels upon pharmacological ALK inhibition using the same ALK-specific tool compound (TAE-684^16,17^) in neuroblastoma cell lines carrying the *ALK* hotspot mutations *ALK*^*R1275Q*^ (CLB-GA) and *ALK*^*F1174L*^ (SH-SY5Y), *ALK* amplification (*ALK*^*amp*^, NB-1) and wild-type *ALK* (*ALK*^*wt*^,SK-N-AS, which harbours the *NRAS*^*Q61K*^ mutation). *ETV5* expression was significantly downregulated in ALK-activated cell lines, while no effect was observed in SK-N-AS (*ALK*^*wt*^, *NRAS*^*Q61K*^) cells (Fig. 1a). Supplementary Fig. S1 shows the endogenous ALK expression levels^18^ and the killing curve of TAE-684. Similar results were obtained with the ALK inhibitor, crizotinib, in CLB-GA and NB-1 cells while this effect was attenuated in SH-SY5Y cells which express the ALK^*F1174L*^ mutant protein. The latter is in keeping with the significant lower responsiveness of this mutation to crizotinib^9^ (Supplementary Fig. S1). The reduction in *ETV5* mRNA levels was confirmed on protein level 6 h after exposure to TAE-684 (Fig. 1b).

In an orthogonal approach, we next tested the effect of enhanced ALK activation by exposure to the ALKAL1 ligand on ETV5 levels in IMR-32 (*ALK*^*wt*^) cells. This caused the expected increased induction of ETV5 protein expression 0.5 h and 6 h after stimulation (Fig. 1c). After stimulation, upregulated p-ALK levels are rapidly attenuated through dephosphorylation in keeping with previously established signalling kinetics^7^. Using artificial stimulation of the ALK receptor with a monoclonal antibody Lopez-Delisle *et al*. also observed an increase in ETV5 mRNA levels after 6 hours^19^. Therefore, our data are in agreement with the activation of ALK leading to induction of expression of ETV5, as clearly shown here at the protein level. The half-life of ETV5 protein after expression induction has not been studied thus far but our findings suggest that the protein is stable up to at least 6 h after the ALK receptor activity itself has been downregulated. We also tested the effect of combined pharmacological ALK inhibition of ALK ligand-stimulated cells which blocked ALK signalling and ETV5 upregulation. This further confirmed ALK-dependent ETV5 regulation (Fig. 1c).

Following these *in vitro* experiments, we assessed the effect of activated ALK on ETV5 expression *in vivo*. Xenograft tumours of SH-SY5Y (*ALK*^*F1174L*^) cells from TAE-684-treated mice showed likewise *ETV5* mRNA downregulation (Supplementary Fig. S1). *ETV5* expression was further also evaluated in concert with *ALK* mutational status in two large independent primary neuroblastoma cohorts in the R2 database^20^ (NRC and GSE49711 datasets). Here, *ETV5* expression was significantly elevated in *ALK*^*mut*^ neuroblastomas compared to tumours harbouring *ALK*^*wt*^ or lacking *ALK* amplifications (Supplementary Fig. S1). This positive correlation thus further supports ALK-dependent *ETV5* regulation. Taken together, both *in vitro* and *in vivo* data further support a role for ETV5 as a target regulated downstream of ALK.

### *ALK* fusion genes upregulate *ETV5* expression in ALCL and NSCLC

Given the importance of activated ALK signalling and since *ALK* fusion genes also achieve enhanced signalling in ALCL and NSCLC, we investigated ALK-induced *ETV5* expression in these cancer entities. We confirmed that in *NPM-ALK*-harbouring Karpas-299 cells (ALCL) and *EML4-ALK*-harbouring H3122 cells (NSCLC) *ETV5* is downregulated upon treatment with the ALK inhibitors, TAE-684 and crizotinib (Fig. 1d). In addition, expression of either ALK mutant proteins (*ALK*^*F1174L*^ and *ALK*^*R1275Q*^) or ALK fusion proteins (*NPM-ALK* and *EML4-ALK*) in Il-3-dependent Ba/F3 pro-B cells caused upregulation of *ETV5* levels (Fig. 1e). Subsequent TAE-684 treatment of Ba/F3 cells dependent on fusion or mutant ALK proteins also reversed *ETV5* expression to the level of the parental Ba/F3 cells, indicating that the observed *ETV5* upregulation is a direct consequence of activated ALK. In summary, our data indicate that *ETV5* regulation by activated ALK is a conserved feature across different cancer and cell types.

### *ALK* regulates ETV5 through the MAPK signalling pathway

To evaluate through which ALK downstream signalling axis *ETV5* is regulated in neuroblastoma cells, we treated CLB-GA (*ALK*^*R1275Q*^), NB-1 (*ALK*^*amp*^), SK-N-AS (*ALK*^*wt*^*, NRAS*^*Q61K*^) and SH-SY5Y (*ALK*^*F1174L*^) cells with inhibitors for the two major pathways downstream of ALK. We used U-0126 as a MAPK inhibitor and pictilisib as a PI_3_K inhibitor^5,21^. Results reveal *ETV5* downregulation only after blocking the MAPK pathway and not the PI_3_K/AKT pathway (Fig. 1f,g and Supplementary Fig. S1**)**. In agreement with earlier reports, SH-SY5Y responds mildly to the MAPK inhibitor U-0126^22–24^, whereas CLB-GA responds more strongly to the PI_3_K/AKT inhibitor pictilisib. This can be explained by compensation by the alternate pathway, since it is known that the RAS/MAPK and PI_3_K/AKT pathways communicate at many nodes^25^. Additionally, both the RET and ALK tyrosine kinase receptors are strongly active in the CLB-GA cell line, which both signal through both pathways (data not shown)^5,26^. Further, as expected, pERK1/2 protein levels were reduced upon pharmacological ALK inhibition in CLB-GA (*ALK*^*R1275Q*^), NB-1 (*ALK*^*amp*^) and SH-SY5Y (*ALK*^*F1174L*^) cells, while levels were unaffected in SK-N-AS (*ALK*^*wt*^*, NRAS*^*Q61K*^) cells (Fig. 1b). Moreover, IMR-32 (*ALK*^*wt*^) cells stimulated with the ALKAL1 *ALK* ligand also showed p-ERK1/2 upregulation in keeping with MAPK activation (Fig. 1c). Our findings indicate that ETV5 is regulated downstream of activated ALK through MAPK-signalling, in line with previous studies reporting ETV5 as a downstream target of RAS/MAPK signalling in other cellular contexts^19,27,28^.

In addition to *ALK* mutations, sequencing studies have also identified activating mutations in other genes upstream of MAPK signalling (including *HRAS, NRAS, KRAS, NF1* and *BRAF*) in neuroblastomas^22^, particularly in relapsed cases. Therefore, we tested the effect of MAPK inhibition using the U-0126 MEK inhibitor in SK-N-AS (*ALK*^*wt*^, *NRAS*^*Q61K*^) cells, in which the *NRAS*^*Q61K*^ mutation resulted in high pERK levels (Fig. 1b, Supplementary Table S1). We observed that SK-N-AS cells were highly sensitivity to MEK inhibition with concomitant strong downregulation of *ETV5* expression (Fig. 1f)^22^. This finding demonstrates that elevated ETV5 levels can result from other upstream MAPK activating events than ALK mutation. To further analyse the relationship between *ETV5* expression and MAPK activation, we investigated *ETV5* expression levels in primary neuroblastomas in the GSE49711 dataset with and without RAS/MAPK pathway mutations (including ALK mutations^29^) (GSE120572). Tumours harbouring RAS/MAPK pathway mutations displayed significantly higher *ETV5* expression. A second analysis in this cohort separating neuroblastomas harbouring either mutant ALK (ALK^mut^) or mutations in other RAS/MAPK pathway genes (RAS/MAPK mut^without ALK^) also revealed significant differences compared to wild-type backgrounds (Fig. 1h). Next, we analysed the correlation between *ETV5* levels and a RAS/MAPK activity score^30^ in a panel of 28 neuroblastoma cell lines and two independent cohorts of 283 and 498 primary neuroblastomas tumours (NRC and GSE49711 datasets, respectively), and found a significant positive correlation (p-values 0.0345, 1.147e-10 and 4.051e-9, respectively, Supplementary Fig. S1). Our findings thus point towards a MAPK pathway-dependent ALK-driven regulation of *ETV5* in neuroblastoma. In line with our findings, Eleveld *et al*.^31^ recently established a 6-gene core RAS/MAPK pathway signature in neuroblastoma that includes *ETV5*. We also confirmed that the remaining genes, ETV1, ETV4, DUSP4 and DUSP6 display similar regulation as *ETV5* upon the inhibition of MAPK, PI_3_K or ALK (Supplementary Fig. S2).

### ETV5 is required for proliferation, migration and colony formation in neuroblastoma cells

**Figure 2:**
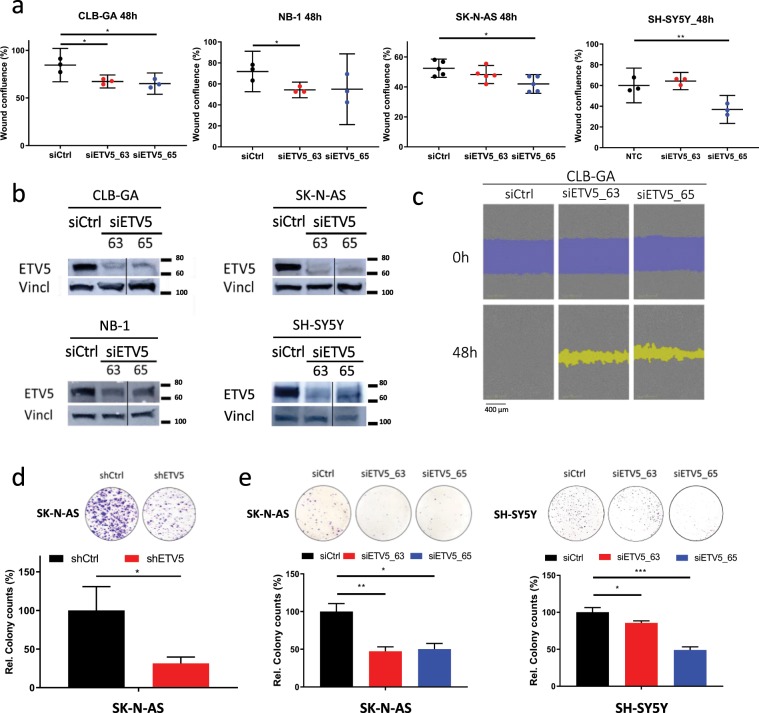
*ETV5* is required for cell migration and colony formation of neuroblastoma cells *in vitro*. (**a**) Wound confluence at 48 h after wound making on IncuCyte® of four different neuroblastoma cell lines after *ETV5* knockdown (siETV5_63 and siETV5_65) compared to control vector (siCtrl). (n_CLB-GA, NB-1, SH-SY5Y_ = 3; n_SK-N-AS_ = 5; mean with error bars representing SD after error propagation). (**b**) Western blot analysis for ETV5 at 48 h after *ETV5* knockdown (siETV5_63 and siETV5_65) in four different neuroblastoma cell lines. (vertical line indicates cropped image, full-length images are presented in Supplementary Fig. S7b). (**c**) Phase contrast imaging of the scratch wound on IncuCyte® for CLB-GA at start (0 h) and after 48 h of *ETV5* knockdown (siETV5_63 and siETV5_65) compared to control vector (siCtrl). Blue represents the initial scratch wound, yellow represents the scratch wound masking. (**d**,**e**) Colony formation analysis of two different NB cell lines after *ETV5* knockdown with shETV5 compared to control vector (shCtrl SHC002; left panel) and after *ETV5* knockdown with siETV5_63 and siETV5_65 compared to control vector (siCtrl; middle and right panel). The upper panel shows the cristal violet staining, the lower panel shows the relative colony counts. (n_shRNA_ = 2; n_siRNA, SK-N-AS_ = 4; n_siRNA, SH-SY5Y_ = 3; mean with error bars representing SD after error propagation) (*p < 0.05; **p < 0.01; ***p < 0.001).

To evaluate the functional impact of ETV5 levels in neuroblastoma cells with different mutant *ALK* backgrounds, we performed transient (siRNA) and stable (shRNA) *ETV5* knockdown in CLB-GA (*ALK*^*R1275Q*^), NB-1 (*ALK*^*amp*^), SK-N-AS (*ALK*^*wt*^*, NRAS*^Q61K^), and SH-SY5Y (*ALK*^*F1174L*^) cells. Migratory capacity was significantly reduced after transient *ETV5* knockdown with either siETV5_63 or siETV5_65 in four different cell lines (Fig. 2a and Supplementary Fig. S3). ETV5 knockdown was confirmed on protein level (Fig. 2b). Further, phase contrast IncuCyte® imaging over 48 h of scratch wound closure illustrated this attenuation in migration (Fig. 2c and Supplementary Fig. S3, QR codes to access movie). In addition, ETV5 knockdown in SK-N-AS (*ALK*^*wt*^*, NRAS*^Q61K^) and SH-SY5Y (*ALK*^*F1174L*^) cells dramatically impaired the ability of the cells to proliferate at limiting density and form colonies (Fig. 2d,e). In view of the previously reported role of ETV5 as a driver for invasion in several tumour types^27^, we also examined the impact of transient *ETV5* knockdown on collagen type I substrate invasion. *ETV5* knockdown in SK-N-AS (*ALK*^*wt*^*, NRAS*^Q61K^) cells significantly reduced the invasive capacity of the cells (Supplementary Fig. S3).

**Figure 3:**
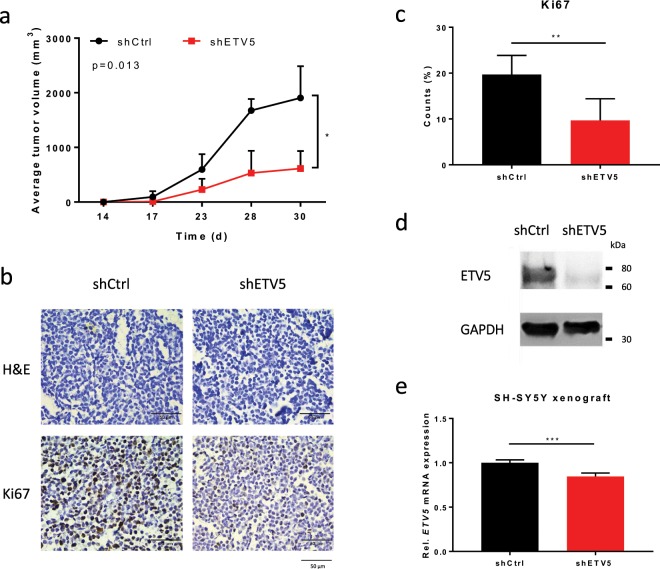
*ETV5* is required for cell growth of neuroblastoma cell lines *in vivo*. (**a**) *In vivo* observation of tumour growth of SH-SY5Y cells after *ETV5* knockdown (shETV5) compared to control cells (shCtrl). (n_shCtrl_ = 4; n_shETV5_ = 6; mean with error bars represent SD after error propagation). (**b**) Histological and immunohistochemical analysis of the xenograft tumour sections with H&E and Ki67 (proliferation) staining. (**c**) Relative Ki67 positive counts (%) for shETV5 xenograft tumours compared to control (shCtrl). (n_shCtrl_ = 5; n_shETV5_ = 6; mean with error bars represent SD after error propagation). (**d**) Western blot analysis for ETV5 after *ETV5* knockdown (shETV5) in SH-SY5Y cells. (cropped images, full-length images are presented in Supplementary Fig. S7c). (**e**) Relative *ETV5* mRNA expression levels of SH-SY5Y xenograft tumour samples after ETV5 knockdown (shETV5) compared to control (shCtrl). (n_shCtrl_ = 4; n_shETV5_ = 5; mean with error bars representing SD after error propagation) (*p < 0.05; **p < 0.01; ***p < 0.001).

The effect of ETV5 on tumour cell behaviour *in vivo* was analysed using the previously used SH-SY5Y xenograft tumour model in immunocompromised mice^32^. These SH-SY5Y cells were transduced with shETV5 to achieve ETV5 knockdown in the xenograft tumours resulting from engraftment. The resulting ETV5 knockdown *in vivo* reduced tumour volume over time compared to tumours originating from the control SH-SY5Y cells (Fig. 3a). Histological examination of these tumours showed a reduction in proliferative activity as measured by Ki-67 staining (Fig. 3b,c). *In vitro* evaluation of the effect of ETV5 knockdown (shETV5) on the cell growth of SH-SY5Y cells showed a significant decrease in proliferative capacity as well (Supplementary Fig. S3). Caspase 3 staining confirmed the exclusion of the involvement of apoptosis in the observed phenotypic effects (data not shown). ETV5 knockdown was confirmed on both RNA and protein levels (Fig. 3d,e**)**. Taken together, our data indicate that ETV5 contributes to proliferation, migration, invasion and colony formation, which can, at least partly, explain the contribution to tumour aggressiveness.

### The *ETV5*-driven transcriptional regulatory network is enriched for genes implicated in proliferation and marks poor patient prognosis

**Figure 4:**
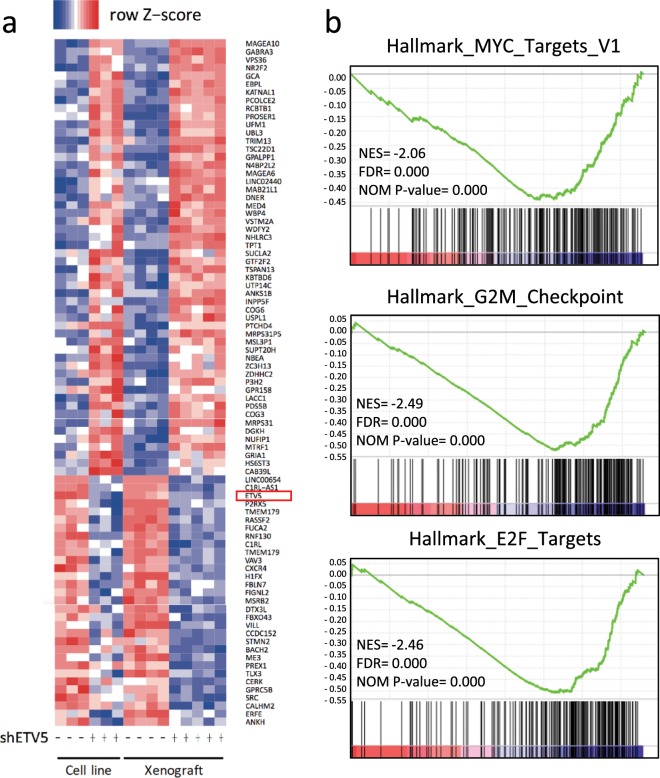
The *ETV5* transcriptional regulatory network controls genes implicated in proliferation. (**a**) Hierarchical clustering and heat-map representation of common significant differentially expressed genes (112 up; 85 down) after *in vitro* and *in vivo ETV5* knockdown in SH-SY5Y. (**b**) Gene Set Enrichment Analysis (GSEA) identifies the hallmark genesets “G2M_CHECKPOINT”, “E2F_TARGETS” and “MYC_TARGETS_V1” as significantly enriched among the downregulated genes after shETV5.

**Figure 5:**
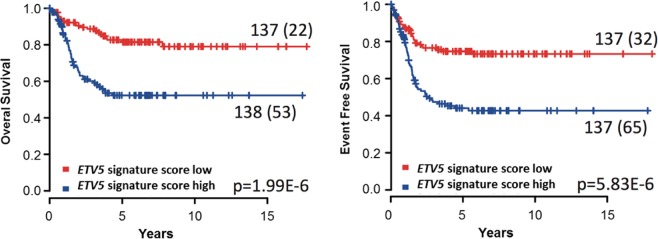
The *ETV5* transcriptional regulatory network marks poor prognosis. *ETV5* activity score, summarizing the expression of 197 genes regulated by *ETV5*, is correlated with worse overall (left panel) and progression-free patient survival (right panel) in the NRC dataset.

To investigate the impact of the ETV5 transcription factor on the neuroblastoma cell transcriptome in relation to the observed phenotypic effects, we performed gene expression profiling after stable *ETV5* knockdown in SH-SY5Y cells *in vitro* and *in vivo* after subcutaneous xenografting. An integrative analysis of these transcriptome datasets generated a total of 197 (112 up and 85 down) significantly differentially expressed genes (Fig. 4a and Supplementary Fig. S4). Gene set enrichment analysis (GSEA)^33^ showed that Hallmark gene sets, “G2/M_CHECKPOINT”, “E2F_TARGETS” and “MYC_TARGETS_V1”, are significantly enriched among genes downregulated after ETV5 knockdown, supporting the observed effect of *ETV5* knockdown on proliferation (Fig. 4b). “APICAL_JUNCTION” (FDR = 0.102; nominal p-value = 0) and “MYOGENESIS” (FDR = 0.187; nominal p-value = 0) were among the top 12 Hallmark gene sets enriched in GSEA, in line with the migratory effects observed *in vitro* (Supplementary Fig. S4). Next, we determined an *ETV5* activity score^34^ summarizing the expression of the 197 genes regulated by *ETV5*. When tested in two independent (transcriptome) datasets from primary neuroblastomas (NRC and GSE49711), the score correlated with worse overall and progression-free patient survival (Fig. 5 and Supplementary Fig. S4)^35,36^. In conclusion, given the enrichment of Hallmark gene sets involved in proliferation and migration, our ETV5 driven transcriptome analysis supports the functional *in vitro* and *in vivo* observations.

## Discussion

The ALK receptor tyrosine kinase plays an important role during normal neuronal development, and constitutive ALK activation has been reported in up to 10% of all neuroblastoma cases^37,38^. Furthermore, activating *ALK* mutations have also been reported to be enriched in relapsed neuroblastoma together with other mutations driving RAS/MAPK signalling^6,22^. Activating *ALK* mutations combined with *MYCN* amplifications in neuroblastoma is associated with very poor prognosis in patients, which is mirrored by the finding that *ALK* mutation accelerates tumour formation in animal models of *MYCN*-driven neuroblastoma^5,10,12^. In view of these findings, a more in-depth understanding of ALK signalling and how downstream target genes impact tumour biology and therapy resistance is of great importance and remained unresolved thus far. For this reason, we previously investigated the transcriptome downstream of *ALK* in neuroblastoma cells and defined a 77-gene signature^5^. Subsequently, we specifically sought *ALK*-upregulated genes implicated in normal neural crest and sympathetic nervous system development with putative oncogenic features and identified *ETV5* as a strong candidate. Earlier reports established ETV5 as a downstream target of the RAS/MAPK pathway in neuroblastoma and other cell types^19,27,28^. We subsequently performed further experiments using pharmacological *ALK* inhibition and ALK ligand stimulation, and confirmed that ALK signalling regulates ETV5 expression levels regardless of whether wild-type or mutant *ALK* is expressed. By exposing neuroblastoma cells to MAPK and PI_3_K/AKT inhibitors, we confirmed that ALK signals through the MAPK pathway to control ETV5 levels. Further in line with our findings, ETV5 was reported to be part of the 6-gene core RAS/MAPK pathway signature specific for neuroblastoma^31^. We also confirm here that the other members of this core signature behave similarly to ETV5 upon treatment with the different inhibitors. Strikingly, ETV5 levels in SK-N-AS (*ALK*^*wt*^*, NRAS*^*Q61K*^) and IMR-32 (*ALK*^*wt*^) were strongly regulated by MAPK inhibition or ALK ligand stimulation, indicating that even in the wild-type ALK background, ETV5 expression plays an important role through MAPK pathway signalling and/or MAPK pathway activating mutations. Further, we extended these findings to other ALKomas, i.e. *EML4-ALK*-harbouring lung cancer and *NPM-ALK*-harbouring lymphomas.

After our extensive validation of ETV5 regulation by ALK and RAS/MAPK signalling in neuroblastoma cells, we investigated the contribution of ETV5 to the neuroblastoma cellular phenotype. ETV5 knockdown *in vitro* and *in vivo* impaired migration, invasion and proliferation of neuroblastoma cells. Our findings corroborate the findings of Hollenhorst *et al*.^39^ and Monge *et al*.^40^, who respectively demonstrated that ETV5 plays a role in prostate cancer cell migration and in the myometrial infiltration of Hec-1A endometrial cancer cells. ETV5 was shown to induce a more aggressive and infiltrative pattern of prostate cancer cells in the former study. ETS factors have also been linked to migration, proliferation and tumour formation in mammary tumours^27,41^. Likewise in bladder cancer, the expression of PEA3 family members were recently shown to be involved in malignant transformation^42^. Our findings that ETV5 knockdown impaired neuroblastoma cell migration and invasive capacity are in line with these findings in multiple tumour entities. We further observed an effect of ETV5 on *in vitro* colony formation and *in vivo* proliferation in neuroblastoma cells, correspondingly as reported by Di Martino *et al*.^42^ in bladder cancer. A decrease in ability to form a compact colony is typical for *Etv4/5* double knockout as well as undifferentiated embryonic cells^43^. Expression of this group of ETS transcription factors has also been correlated to expression of certain matrix metalloproteinases (MMP), amongst other molecules, which are known to degrade extra-cellular matrix components^41^. The precise molecular principle by which ETV5 impairs neuroblastoma cell migration and invasion remains, however, to be elucidated.

Our finding that ETV5 affects neuroblastoma cell migration, invasive and colony formation capacity and ETV5 upregulation through MAPK signalling (either through activated ALK or RAS pathway activation) suggests that ETV5 may contribute to increased aggressiveness and relapse of neuroblastoma through darwinistic selection of ALK/RAS/MAPK pathway activating mutations during therapy. This is further in keeping with the *ETV5*-driven gene signature established in this study which also correlated with poor survival in patients with neuroblastoma. Additional studies, including orthotopic *in vivo* modelling, are however needed to further investigate and clarify the possible contribution of ETV5 to aggressive tumour cell behaviour. In this context, it is of interest that ETV5, together with ETV4, has been implicated in stemness of embryonic stem cells by promoting their naïve status^43^, as well as the terminal differentiation and neurite branching of dorsal root ganglion sensory neurons^44^ and, via induction by the BDNF/TrkB pathway, in neuronal outgrowth^45^. In embryonic stem cells, *ETV5* has been proposed to be part of a large gene regulatory network of transcription factors in which *OCT3/4* and *NANOG* act as master regulators^43^.

Drug resistance remains a major challenge in the era of precision cancer treatment. The finding by Umapathy *et al*. that ALK-activated neuroblastoma cells are resistant to the MEK inhibitor, trametinib, due to PI_3_K/AKT-driven feed-back response is of interest^46^ with regards to the known crosstalk between the MAPK and PI_3_K/AKT pathways. Together with our findings, this work further illustrates the importance of in-depth knowledge of context-dependent signalling in oncogene-activated pathways and how they respond through rewiring upon drug targeting. At present, we can only speculate on a potential role for ETV5 in trametinib resistance in ALK-activated neuroblastoma. *ETV5* levels were concomitantly downregulated in SK-N-AS cells in the course of their strong response to MEK inhibition in our experiments. These neuroblastoma cells harbour the activating *NRAS*^*Q61K*^ mutation in a wild-type *ALK* cellular background. In ALK-activating neuroblastoma cellular backgrounds, the effects of *ETV5* reduction on MEK inhibition were significant, albeit attenuated when compared to the same cell lines treated with *ALK* inhibitor. The negative MAPK regulator DUSP6 is previously shown to be regulated by ETV5 in zebrafish^47^, which could explain our previous finding of a MAPK negative feed-back loop in ALK-activated neuroblastoma cells^5^. As mentioned earlier, other members of the RAS/MAPK pathway signature in neuroblastoma^31^ behaved similarly to ETV5, albeit less pronounced, upon treatment with the different inhibitors. These data thus suggest that ETV5 could be an important driver of this negative feed-back loop.

*ETV5* and other PEA3 family members, as well as several other ETS factors, are widely implicated in various cancer entities such as prostate, Ewing and melanoma tumours^27,48^. Neuroblastomas and most likely other ALKomas can now be added to this growing list. Our finding implicating ETV5 in neuroblastoma cell migration, proliferation and invasion is in agreement with ETV5 overexpression causing enhanced proliferation and invasion in immortalized, non-transformed prostate epithelial cells^48–50^ and ETV5 functional inhibition attenuating proliferation in mouse mammary cancer^27^. The broad implication of these transcription factors in cancer, creates a growing need for ETS transcription factor targeted therapy. In order to abolish ETV5 activity, possible approaches include emerging methods to target protein degradation^51^, interference with functional binding partners and drugging key target genes or upstream activators such as MAPK signalling. Recent discoveries in the development of small molecules targeting ETS proteins raise hope^52^. Therefore, focussing on targeting ETV5 activity could serve as a basis for new complementary or synergistic therapies oriented to targeting effectors downstream of ALK.

## Materials and Methods

### Cell lines and inhibitors

Cell lines used, with their respective mutations, are listed in Supplementary Table S1. The murine interleukin 3 (Il-3)-dependent pro-B-Cell line, Ba/F3, was used as a model to obtain *ALK*-dependent cells, owing to its potent growth capacity and use of kinase oncogene signalling^53^. Cells were grown in RPMI 1640 (Invitrogen) supplemented with 10% FCS, 100IU/ml penicillin/streptomycin, 2 mM L-glutamine and 25 mM HEPES (Life Technologies). Ba/F3 cells were cultured in the presence of 1 ng/mL Il-3 (213-13, Peprotech). Cells were cultured at 37 °C in a 5% CO_2_/95% O_2_ humidified environment. Mycoplasma testing (Lonza) and short tandem repeat genotyping were regularly performed.

Cells were treated for indicated time periods with inhibitory compounds: the ALK inhibitors, NVP-TAE-684 (0.32 µM, S1108, Novartis/SelleckChem), crizotinib (0.5 µM, PF-02341066, S1068, Pfizer/SelleckChem) or LDK-378 (0.2 µM, S7083, SelleckChem); the MEK inhibitors, U-0126 (8 µM, Sigma-Aldrich) or trametinib (0.05 µM, GSK1120212, S2673, SelleckChem); the dual PI_3_K/mTOR inhibitor, NVP-BEZ-235 (0.5 µM, S1009_3, SelleckChem) or the PI_3_K inhibitor pictilisib (0.5 µM, S1065, SelleckChem). Dimethyl sulfoxide (DMSO, VWR) was used to dissolve the compounds and served as vehicle control.

### *ALK* constructs, siETV5 transfections/nucleofections and shETV5 transductions

Expression constructs for the F1174L and R1275Q ALK mutant proteins were generated as described in De Brouwer *et al*.^3^.

*ETV5* knockdown used siRNA oligonucleotides from ON-TARGET plus SMARTpools (L-062952-01-0005, Dharmacon, referred to as siETV5) and Lipofectamine^TM^ RNAiMAX transfection Silencer® Select reagents (4392420-s4863 and –s4865, Fisher Scientific, referred to as siETV5_63 and siETV5_65 respectively). As a control, siCONTROL non-targeting (siCtrl) siRNA pool (D-001810-10-05, Dharmacon) and Negative Control #1 siRNA (1299001, Fisher Scientific) were used. CLB-GA, NB-1 and SK-N-AS cells were transfected according to manufacturer’s instructions. SH-SY5Y cells were nucleofected with 100 nM of the above described siRNAs using the Neon Transfection System (Thermo Fisher Scientific).

Five *ETV5* mission shRNAs were obtained from the TRC1 library (Sigma-Aldrich, TRCN0000013938 till −42). TRCN0000013938 (referred to as shETV5), was used throughout this manuscript as the optimal shRNA (target sequence: CCGGCCGTGACACTTAGTACATTAACTCGAGTTAATGTACTAAGTGTCACGGTTTTT). Lentiviral production was according to the Thermo Scientific Trans-Lentiviral Packaging Kit (TLP5913). Transduced SH-SY5Y and SK-N-AS cells were selected using 0.5–1 µg/ml puromycin (P8833, Sigma-Aldrich). The transduction efficiency was determined by qPCR and western blotting. pLKO.1-puro Non-Mammalian shRNA control plasmid (SHC002, target sequence: CCGGCAACAAGATGAAGAGCACCAACTCGAGTTGGTGCTCTTCATCTTGTTGTTTTT, Sigma-Aldrich) was used as a negative control.

### ALKAL1 ligand for *ALK* stimulation

The ALKAL1 *ALK* ligand was used to stimulate ALK signalling in cell culture by treatment with conditioned medium as previously described by Guan *et al*.^7^.

### Reverse-Transcriptase quantitative Polymerase Chain Reaction (RT-qPCR)

RT-qPCR was performed on samples as previously described^5^. Expression levels of target genes were normalized to a minimum of three internal reference genes. All primer sequences were acquired by the use of RTPrimerDB (http://www.rtprimerdb.org) (Supplementary Table S2)^54^. Expression analysis and error propagation was done using qBasePLUS software 1.5 (http://www.biogazelle.com)^55^. Error bars in figures represent SD or 95% CI after error propagation with mean centring and scaling to control. For statistical testing, a paired two-tailed Student’s t-test was performed at the 5% significance level.

### Protein analysis

Protein isolation and western blotting was performed as previously described^5^. The following primary antibodies were used: anti-pY1604-ALK (1:500, 3341), anti-ALK (1:750, 3333), anti-pERK1/2 (1:500, 9101S) and anti-ERK1/2 (1:500, 9102), all from Cell Signaling; anti-ETV5 from Abnova (1:500, H00002115-M01). For the loading control, anti-β-actin (Cell Signaling), anti-vinculin (Sigma Aldrich) and anti-GAPDH (Genetex) antibodies were used. Secondary antibodies were used from Cell Signaling. Imaging was done using the Amersham Imager 680 (GE Healthcare).

### Cell migration assays

Neuroblastoma cells were seeded for IncuCyte® scratch wound assay, while protein samples were simultaneously harvested from equivalent cell aliquots. Cells were seeded in an Imagelock 96-well plate (4379, Essen Bioscience). Uniform wounds were made in the confluent cell monolayer using the Incucyte® Wound Maker, and cells were re-transfected according to manufacturer’s instructions. Phase contrast imaging took place every 2 h for a total of 48 h. Images were analysed using IncuCyte® S3^TM^ 2018B-2019A software. Error bars in figures represent SD after error propagation with mean centring and scaling to control. For statistical testing, a Levine’s test was performed in SPSS at the 1% significance level upon which an independent paired t-test was performed at the 5% significance level^56,57^.

### Colony formation assay

SK-N-AS cells were seeded at a concentration of 2000 cells per 6 cm dish, 5000 SH-SY5Y cells were seeded per well of a 6-well plate. Cells were seeded in 2 or 3-fold for experiments. Subsequently colonies were allowed to form followed by fixation as previously described^13^. OpenCFU was used to quantify differences in colony forming capacity. Error bars in figures represent SD after error propagation. For statistical testing, a paired two-tailed Student’s t-test was performed at the 5% significance level.

### Collagen invasion assay

The collagen invasion assay of SK-N-AS cells after siETV5 transfection was performed as previously described by De Wever *et al*.^58^. Error bars in figures represent SD after error propagation. For statistical testing, a Chi² test was performed at the 5% significance level.

### Cell proliferation assays

SH-SY5Y were transduced with shETV5 lentiviral particles as described above. Upon 6 days of puromycin selection, cells were seeded for IncuCyte® proliferation assay, while samples for protein and RNA were harvested simultaneously from equivalent cell aliquots. Cells were seeded in triplicate in a 96-well plate (3596, Corning). Phase contrast imaging took place every 2 h for a total of 112 h. Images were analysed using the IncuCyte® Zoom^TM^ 2016B software. Error bars in figures represent SD after error propagation. For statistical testing, a paired two-tailed Student’s t-test was performed at the 5% significance level.

### Xenograft experiments

Animal experiments were performed according to the *Guide for the Care and Use of Laboratory Animals* (Eight Edition) following approval of the Committee on Ethics in Animal Experiments of Ghent University (Permit number: ECD 12/22)^59^. Persons who carried out the described experiments received appropriate training in animal care and handling. Mice were allowed to acclimatize before experiments began and were randomly assigned into two groups.

6 × 10^6^ control SH-SY5Y or SH-SY5Y shETV5 cells were mixed with Matrigel® (354230, Corning) and subsequently injected subcutaneously into the left flank of female Crl:NU-Foxn1nu mice. Tumour growth was followed for 33 days. Tumour volume was assessed using a Caliper and calculated according to the spheroid formula: **V** = 0.5*a²*b with **a** being the smallest and **b** being the largest superficial diameter. Body weight and physical status of the animals were recorded daily until they were judged to be in discomfort by animal caretakers or maximum tumour volume was reached (2000 mm³) where after the animal was euthanized by cervical dislocation.

*ETV5* mRNA levels were further evaluated upon treatment with TAE-684 of mice subcutaneously xenografted with SH-SY5Y as described by Heukamp *et al*.^60^.

### Immunohistochemistry

Xenograft tumours were fixed overnight in 4% paraformaldehyde, before dehydrating and embedding in paraffin. 5 µm sections were stained with H&E (Sigma). Antigen retrieval was carried out in citrate buffer (S236984, Dako) and endogenous peroxidases were blocked with 3% H_2_O_2_ in methanol (0390D, Fisher Chemical). Sections were incubated with anti-Ki67 (ab15580, Abcam) primary antibody and stained with biotin-conjugated secondary antibody followed by streptavidin-HRP-based DAB substrate development (K3468, Dako). Images were acquired with the 40x objective on a Leica DM2000 LED microscope and processed with the LAS-X software. Images were evaluated by Fiji (ImageJ). Error bars of figures represent SD after error propagation. For statistical testing, a paired two-tailed Student’s t-test was performed at the 5% significance level.

### Differential gene expression analysis by RNA sequencing

RNA sequencing was carried out on biological triplicates for shETV5-based knockdown experiments and on matched samples of subsequently generated mice xenograft tumours. Libraries for mRNA sequencing were prepared using the TruSeq stranded mRNA sample prep kit (Illumina), involving polyA-selection, fragmentation, adapter ligation, reverse transcription and PCR amplification. Libraries were quantified on a Qubit 2.0 Fluorometer prior to paired-end sequencing with 75 bp read length on a NextSeq. 500 sequencer (Illumina). On average 24.7 M high-quality reads were generated per sample, with a minimal read count of 20.0 M reads per sample. For each sample, gene-level read counts were generated using Sailfish^61^. Counts were normalized using the TMM method (R-package edgeR), followed by Voom transformation and differential expression analysis with limma (R-package limma). Gene Set Enrichment Analysis (GSEA)^33^ was performed on the list ordered according to the differential expression statistic value (t) using the version 5.0 Hallmarks geneset catalogue. RNA sequencing data is available at ArrayExpress (E-MTAB-6713).

### Signature score analysis in primary neuroblastomas

Two expression datasets were reanalysed, a partly published NRC dataset containing mRNA expression data (Affymetrix Exon arrays) from 283 neuroblastoma samples^35,62,63^ and the GSE49711 dataset including RNA sequencing data from 498 neuroblastomas that includes a subset of 259 neuroblastomas with defined RAS/MAPK mutation status (GSE120572)^29,36,64^. The RAS/MAPK signature was retrieved from the Loboda *et al*. study^30^.

Signature score analysis was performed on these expression data using a rank-scoring algorithm as described by Fredlund *et al*.^34^. Correlation with survival was tested using Kaplan-Meier plots and log-rank analysis by grouping samples into 2 equal groups (scores above or below the median value) (R-survival package).

### Statistical analysis

The statistical analysis performed are conform to each specific assay. Therefore it was chosen to describe the specific statistical analysis at the end of each assay description mentioned above.

## Supplementary information

Supplementary Information.
