# Supplementary material for: The ETS transcription factor ETV5 is a target of activated ALK in neuroblastoma contributing to increased tumour aggressiveness
Source: Sci Rep. 2020 Jan 14;10:218. doi: 10.1038/s41598-019-57076-5 (PMC6959226; doi:10.1038/s41598-019-57076-5)
Supplement: Supplementary file 1 — Supplementary Information. [file 41598_2019_57076_MOESM1_ESM.pdf]

Supplemental Info

The ETS transcription factor ETV5 is a target of activated ALK in neuroblastoma contributing to increased tumour aggressiveness

Liselot M. Mus[1,2], Irina Lambertz[1,2], Shana Claeys[1,2], Candy Kumps[3], Wouter Van Loocke[1,2], Christophe Van Neste[1,2], Ganesh Umapathy[4], Marica Vaapil[5], Christoph Bartenhagen[6,7], Genevieve Laureys[1,8], Olivier De Wever[2,9], Daniel Bexell[5], Matthias Fischer[6,7], Bengt Hallberg[4], Johannes Schulte[10,11,12,13], Bram De Wilde[1,2,8], Kaat Durinck[1,2], Geertrui Denecker[1,2], Katleen De Preter[1,2], Frank Speleman[1,2] *

[1] Department of Biomolecular Medicine, Ghent University, Ghent, Belgium
[2] Cancer Research Institute Ghent (CRIG), Ghent, Belgium
[3] Department of Uro-gynaecology, Ghent University Hospital, Ghent, Belgium
[4] Department of Medical Biochemistry and Cell Biology, Institute of Biomedicine, Sahlgrenska Academy, University of Gothenburg, Gothenburg, Sweden
[5] Translational Cancer Research, Lund University, Lund, Sweden
[6] Department of Experimental Pediatric Oncology, University Children's Hospital of Cologne, Medical Faculty, University of Cologne, 50937, Cologne, Germany
[7] Centre for Molecular Medicine Cologne (CMMC), University of Cologne, 50931, Cologne, Germany
[8] Department of Paediatric Haematology and Oncology, Ghent University Hospital, Ghent, Belgium
[9] Laboratory of Experimental Cancer Research, Ghent University, Belgium
[10] Department of Paediatric Oncology and Haematology, University Children's Hospital Essen, Germany
[11] Department of Paediatric Oncology and Haematology, Charité University Medical Centre Berlin, Berlin, Germany
[12] German Cancer Consortium (DKTK), Berlin, Germany
[13] German Cancer Research Centre (DKFZ), Heidelberg, Germany
* Corresponding author

SUPPLEMENTARY TABLE LEGENDS

Supplemental Table S1: Cell line information. Summary of the characteristics of the cell lines used in the study including their origin, the type of tumour, their *ALK* and *MYCN* mutation status.

Supplemental table S1

Sample ID	Origin	Tumour	ALK status	MYCN status
CLB-GA	Combaret	NB	R1275Q	NA
IMR-32	Versteeg	NB	wt	A
NB-1	JHSF	NB	wt (ampl)	A
SH-SY5Y	Schulte	NB	F1174L	NA
SK-N-AS	ATCC	NB	wt	NA
Karpas-299	/	ALCL	NPM-ALK fusion gene	/
H3122	/	NSCLC	EML4-ALK fusion gene	/
Ba/F3	/	Murine pro-B cell	/	/

NB = neuroblastoma, ALCL = anaplastic large cell lymphoma, NSCLC = non-small cell lung cancer, wt = wild type, NA = non amplified, A = amplified

Supplemental Table S2: qPCR primer sequences. Full primer sequences used for target and reference genes during RT-qPCR.

Supplemental table S2

Gene name	Species	Forward primer	Reverse primer
ETV5	human	AGCTCTGCAGAATCGTGAG	TCTCGATCTGAGGAATGCAG
ETV1	human	GAACCCCACAGTCCATGTTC	CATTCCCACTTGTGGCTTCT
ETV4	human	TGGTGATCAAACAGGAACAG	CTCTGTGTGGAGGTACATTG
DUSP4	human	AGGAGGAAGAAAGGGAAGAA	CCTTGGCAACATAGTGAGAT
DUSP6	human	AGCTCAATCTGTCGATGAAC	CTGACCCATGAAGTTGAAGT
TBP	human	CACGAACCACGGCACTGATT	TTTTCTTGCTGCCAGTCTGGAC
YWHAZ	human	ACTTTTGGTACATTGTGGCTTCAA	CCGCCAGGACAAACCAGTAT
HPRT1	human	TGACACTGGCAAAACAATGCA	GGTCCTTTTCACCAGCAAGCT
UBC	human	ATTTGGGTCGCGGTTCTTG	TGCCTTGACATTCTCGATGGT
Etv5	mouse	GCTCACGATTCTGAAGAGTT	GCTCATCATCAGGAACTTGT
Hprt1	mouse	CCTAAGATGAGCGCAAGTTGAA	CCACAGGACTAGAACACCTGCTAA

SUPPLEMENTAL FIGURE LEGENDS

Supplemental figure S1: ETV5 expression is regulated by ALK through the MAPK signalling pathway. a. Endogenous relative expression levels of *MYCN, ALK* and *ETV5* in six different neuroblastoma cell lines[18] (arrows indicate values exceeding y-axis limits). **b.** Killing curve of TAE-684 on six different neuroblastoma cell lines. (n=3; mean with error bars representing SD after error propagation) **c.** Relative *ETV5* expression levels in four different neuroblastoma cell lines 6h post-treatment with a vehicle control (DMSO) or the ALK inhibitors crizotinib (0.5µM). (n=4: mean with error bars representing 95% CI after error propagation with mean centring and scaling to control) **d.** Relative *ETV5* mRNA expression levels in TAE-684 treated SH-SY5Y xenograft tumours compared to control. (n=2; mean with error bars representing SD after error propagation) **e.** Boxplot representation of the log2 *ETV5* mRNA expression levels in two large independent cohorts of primary neuroblastomas in the R2 database (NRC and GSE49711 datasets) in ALK^{mut} versus ALK^{wt} tumours. **f.** Log2 *ETV5* mRNA expression levels in CLB-GA after 6h of MEK (trametinib, 0.05µM), PI_3K (BEZ-235, 0.5µM) or ALK (crizotinib, 0.5µM; LDK-378, 0.2µM and TAE-684, 0.3µM) inhibition. (n=2; mean with error bars representing SD after error propagation) **g.** Correlation plot of *ETV5* mRNA expression levels and the RAS activity score in a panel of 28 neuroblastoma cell lines and two independent cohorts of 283 and 498 primary neuroblastomas in R2 (NRC and GSE49711 datasets respectively). (* p<0.05; ** p<0.01; ***p<0.001)

Supplemental figure S2: RAS-MAPK signature gene expression in neuroblastoma cells according to Eleveld *et al.*, 2018 follow the ETV5 expression levels upon compound inhibition. a. Relative *ETV1, ETV4, DUSP4 and DUSP6* expression levels in four different neuroblastoma cell lines upon a time series treatment with a vehicle control (DMSO) or the ALK inhibitor TAE-684 (0.3 µM). (n_{CLB-GA}=3; n_{NB-1}= 4; $n_{SK-N-AS}$=3; $n_{SH-SY5Y}$=5; mean with error bars representing 95% CI upon error propagation with mean centring and scaling to control) **b-d.** Relative *ETV1, ETV4, DUSP4 and DUSP6* expression levels in four different neuroblastoma cell lines upon 6h treatment with a vehicle control (DMSO) or the MEK inhibitor U-0126 (8µM), the PI_3K inhibitor pictilisib (500nM) or the ALK inhibitors crizotinib (0.5µM) respectively. (n_{CLB-GA}=3; $n_{NB-1, pictilisib, ETV1}$=6; $n_{NB-1, pictilisib, ETV4, DUSP6}$=5; $n_{NB-1, pictilisib, DUSP4}$=3; $n_{NB-1, U-0126}$=3; $n_{NB-1, crizotinib, ETV1, ETV4, DUSP4}$=4; $n_{NB-1, crizotinib, DUSP6}$=2; $n_{SK-N-AS}$=3; $n_{SH-SY5Y, pictilisib, ETV1, DUSP6}$=4; $n_{SH-SY5Y, pictilisib, ETV4, DUSP4}$=5; $n_{SH-SY5Y, U-0126}$=5; $n_{SH-SY5Y, crizotinib, ETV1, DUSP6}$=4; $n_{SH-SY5Y, crizotinib, ETV4, DUSP4}$=5; mean with error

bars representing 95% CI upon error propagation with mean centring and scaling to control) (* $p<0.05$; ** $p<0.01$; ***$p<0.001$)

Supplemental figure S3: ETV5 is required for cell migration and invasion of neuroblastoma cell lines. a. Relative difference in wound confluence (%) as measured on IncuCyte® given every 2h over a total of 48h for four different neuroblastoma cell lines after *ETV5* knockdown (siETV5_63 and siETV5_65) compared to control vector (siCtrl). ($n_{CLB\text{-}GA,\ NB\text{-}1,\ SH\text{-}SY5Y}=3$; $n_{SK\text{-}N\text{-}AS}=5$; mean with error bars representing SD after error propagation with mean centring and scaling to control) **b.** Levine's test (robust test of equality of means) was performed at the 1% significance level upon which an independent paired t-test was performed at the 5% significance level for the wound confluence. Results are given every 2h over a total of 48h for four different neuroblastoma cell lines after *ETV5* knockdown (siETV5_63 and siETV5_65) compared to control vector (siCtrl). **c.** Phase contrast movie of CLB-GA upon *ETV5* knockdown (siETV5_63 and siETV5_65) compared to control vector (siCtrl). Yellow represents the scratch wound masking. Scan QR codes to access movie. **d.** Collagen invasion assay count (%) of SK-NAS cells after *ETV5* knockdown (siETV5) or control vector (siCtrl). (n=5; mean with error bars representing SD after error propagation) **e.** Relative proliferation of SH-SY5Y cells after ETV5 knockdown (shETV5) compared to control (shCtrl). (n=3) (* $p<0.05$; ** $p<0.01$; ***$p<0.001$)

Supplemental figure S4: The ETV5 transcriptional regulatory network controls genes implicated in proliferation, migration and marks poor prognosis. a. Volcano plot representation of significant differentially expressed genes (112 up; 85 down) upon *in vitro* and *in vivo ETV5* knockdown in SH-SY5Y. **b.** Gene Set Enrichment Analysis (GSEA) identifies the hallmark genesets “APICAL_JUNCTION” and “MYOGENESIS” as enriched among the downregulated genes upon shETV5. **c.** *ETV5* activity score, summarizing the expression of the 197 genes regulated by *ETV5*, is correlated with worse overall survival (left panel) and progression-free patient survival (right panel) in the GSE49711 dataset.

Supplemental figure S5: Full western blot images Fig. 1b ALK regulates ETV5 expression through the MAPK signalling pathway. Western blot analysis for p-ALK and total ALK in four different neuroblastoma cell lines after ALK inhibition with TAE-684 (0.3µM, 6h).

Supplemental figure S6: Full western blot images Fig. 1b ALK regulates ETV5 expression through the MAPK signalling pathway. Western blot analysis for ETV5, p-ERK1/2 and total ERK1/2 in four different neuroblastoma cell lines after ALK inhibition with TAE-684 (0.3µM, 6h).

Supplemental figure S7: Full western blot images Fig. 1c ALK regulates ETV5 expression through the MAPK signalling pathway, Fig. 2b *ETV5* is required for cell migration and colony formation of neuroblastoma cells *in vitro* and Fig. 3d *ETV5* is required for cell growth of neuroblastoma cell lines *in vivo.* a. Western blot analysis for p-ALK, total ALK, ETV5 and p-ERK1/2 in IMR-32 after ALK ligand (ALKAL1) stimulation for 30min or 6h and subsequent treatment with ALK inhibitor, crizotinib (0.25µM). **b.** Western blot analysis for ETV5 at 48h after *ETV5* knockdown (siETV5_63 and siETV5_65) in four different neuroblastoma cell lines. **c.** Western blot analysis for ETV5 after *ETV5* knockdown (shETV5) in SH-SY5Y neuroblastoma cells.

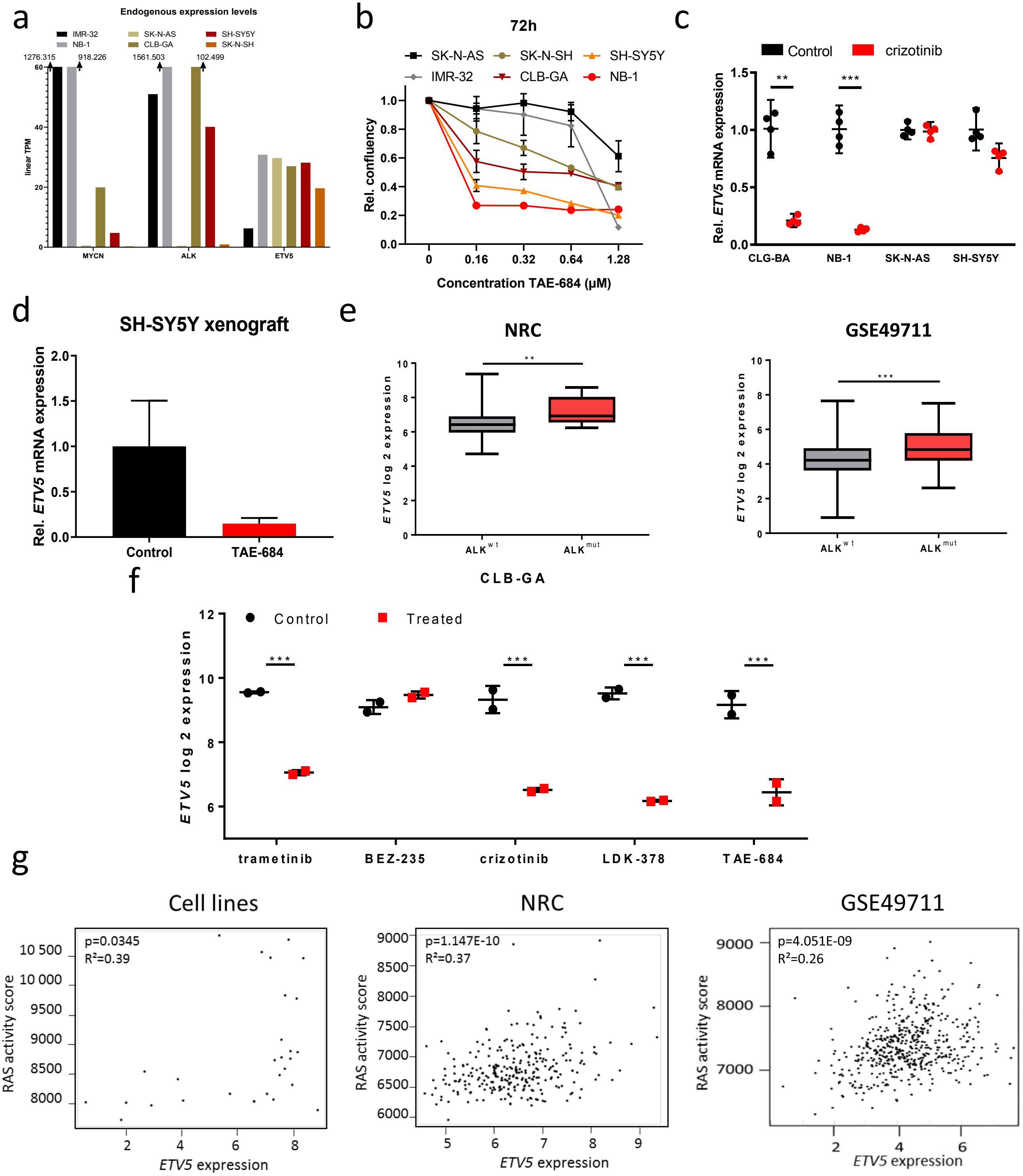
a
Endogenous expression levels
IMR-32
NB-1
SK-N-AS
CLB-GA
SH-SY5Y
SK-N-SH
1276.315
918.226
1561.503
102.499
linear TPM
MYCN
ALK
ETV5
b
72h
SK-N-AS
SK-N-SH
SH-SY5Y
IMR-32
CLB-GA
NB-1
Rel. confluency
Concentration TAE-684 (μM)
c
Control
crizotinib
Rel. ETV5 mRNA expression
CLG-BA
NB-1
SK-N-AS
SH-SY5Y
d
SH-SY5Y xenograft
Rel. ETV5 mRNA expression
Control
TAE-684
e
NRC
GSE49711
ETV5 log 2 expression
ALK wt
ALK mut
CLB-GA
f
Control
Treated
ETV5 log 2 expression
trametinib
BEZ-235
crizotinib
LDK-378
TAE-684
g
Cell lines
NRC
GSE49711
p=0.0345
R²=0.39
p=1.147E-10
R²=0.37
p=4.051E-09
R²=0.26
RAS activity score
ETV5 expression

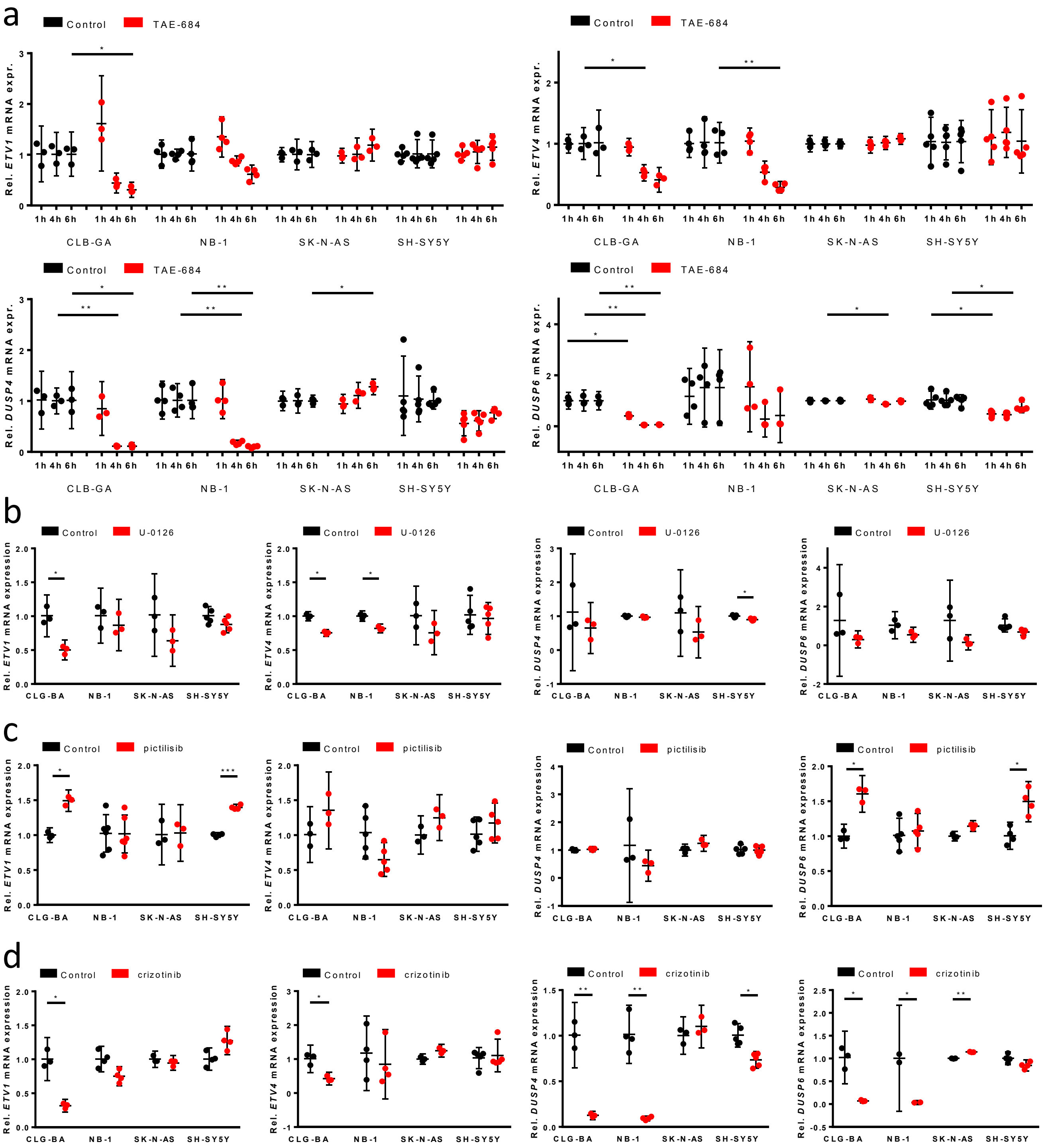
a
Control
TAE-684
Rel. ETV1 mRNA expr.
Rel. ETV4 mRNA expr.
Rel. DUSP4 mRNA expr.
Rel. DUSP6 mRNA expr.
1h 4h 6h
CLB-GA
NB-1
SK-N-AS
SH-SY5Y
b
Control
U-0126
Rel. ETV1 mRNA expression
Rel. ETV4 mRNA expression
Rel. DUSP4 mRNA expression
Rel. DUSP6 mRNA expression
CLG-BA
NB-1
SK-N-AS
SH-SY5Y
c
Control
pictilisib
Rel. ETV1 mRNA expression
Rel. ETV4 mRNA expression
Rel. DUSP4 mRNA expression
Rel. DUSP6 mRNA expression
CLG-BA
NB-1
SK-N-AS
SH-SY5Y
d
Control
crizotinib
Rel. ETV1 mRNA expression
Rel. ETV4 mRNA expression
Rel. DUSP4 mRNA expression
Rel. DUSP6 mRNA expression
CLG-BA
NB-1
SK-N-AS
SH-SY5Y

a

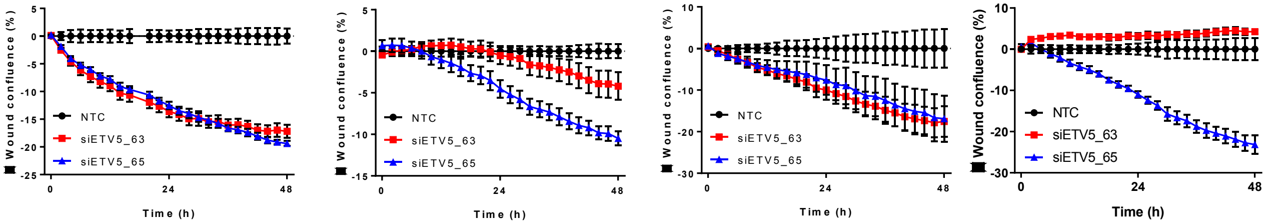

b

CLB-GA

Time point (h)	Levene's test statistic	t-test p value NTC vs siETV5_63	Levene's test statistic	t-test p value NTC vs siETV5_65
0	0,532	0,694	0,674	0,677
2	0,354	0,217	0,372	0,345
4	0,338	0,228	0,334	0,283
6	0,386	0,216	0,42	0,285
8	0,352	0,192	0,492	0,286
10	0,409	0,179	0,523	0,247
12	0,396	0,17	0,458	0,23
14	0,366	0,12	0,578	0,185
16	0,336	0,131	0,555	0,191
18	0,384	0,28	0,773	0,435
20	0,479	0,123	0,65	0,181
22	0,43	0,107	0,65	0,155
24	0,483	0,085	0,69	0,126
26	0,453	0,073	0,72	0,104
28	0,426	0,058	0,76	0,093
30	0,375	0,062	0,725	0,087
32	0,422	0,057	0,686	0,075
34	0,384	0,049	0,713	0,061
36	0,441	0,045	0,738	0,053
38	0,354	0,041	0,636	0,046
40	0,352	0,034	0,589	0,035
42	0,313	0,027	0,529	0,028
44	0,286	0,023	0,515	0,023
46	0,285	0,023	0,526	0,021
48	0,267	0,017	0,534	0,016

SK-N-AS

Time point (h)	Levene's test statistic	t-test p value NTC vs siETV5_63	Levene's test statistic	t-test p value NTC vs siETV5_65
0	0,048	0,664	0,393	0,658
2	0,105	0,929	0,296	0,672
4	0,111	0,93	0,212	0,712
6	0,028	0,875	0,232	0,851
8	0,042	0,665	0,183	0,998
10	0,028	0,698	0,27	0,779
12	0,075	0,721	0,347	0,695
14	0,1	0,74	0,513	0,605
16	0,133	0,825	0,554	0,514
18	0,146	0,908	0,908	0,40
20	0,172	0,927	0,819	0,38
22	0,243	0,975	0,654	0,31
24	0,242	0,866	0,926	0,22
26	0,244	0,841	0,873	0,181
28	0,284	0,798	0,849	0,149
30	0,37	0,634	0,853	0,1
32	0,339	0,61	0,833	0,086
34	0,29	0,577	0,764	0,079
36	0,287	0,523	0,76	0,061
38	0,397	0,47	0,807	0,039
40	0,428	0,391	0,884	0,031
42	0,417	0,318	0,805	0,025
44	0,473	0,242	0,878	0,015
46	0,471	0,247	0,818	0,015
48	0,663	0,211	0,95	0,01

NB-1

Time point (h)	Levene's test statistic	t-test p value NTC vs siETV5_63	Levene's test statistic	t-test p value NTC vs siETV5_65
0	0,408	0,768	0,848	0,57
2	0,432	0,645	0,139	0,294
4	0,26	0,247	0,99	0,236
6	0,232	0,087	0,532	0,235
8	0,056	0,056	0,378	0,222
10	0,044	0,023	0,246	0,188
12	0,06	0,019	0,238	0,19
14	0,057	0,021	0,33	0,193
16	0,054	0,03	0,322	0,248
18	0,042	0,02	0,222	0,28
20	0,03	0,02	0,199	0,30
22	0,049	0,016	0,211	0,29
24	0,076	0,019	0,195	0,28
26	0,094	0,021	0,25	0,249
28	0,075	0,022	0,225	0,284
30	0,086	0,019	0,297	0,234
32	0,075	0,021	0,267	0,216
34	0,081	0,022	0,304	0,221
36	0,118	0,022	0,331	0,211
38	0,1	0,017	0,293	0,179
40	0,116	0,015	0,302	0,167
42	0,104	0,018	0,314	0,171
44	0,138	0,019	0,377	0,161
46	0,112	0,019	0,362	0,148
48	0,153	0,022	0,419	0,134

SH-SY5Y

Time point (h)	Levene's test statistic	t-test p value NTC vs siETV5_63	Levene's test statistic	t-test p value NTC vs siETV5_65
0	0,505	0,972	0,039	0,639
2	0,532	0,736	0,472	0,833
4	0,668	0,671	0,531	0,997
6	0,693	0,613	0,576	0,868
8	0,948	0,582	0,608	0,748
10	0,967	0,533	0,738	0,574
12	0,842	0,585	0,787	0,502
14	0,663	0,583	0,927	0,43
16	0,635	0,556	0,933	0,321
18	0,426	0,566	0,701	0,22
20	0,394	0,562	0,788	0,18
22	0,444	0,517	0,802	0,132
24	0,375	0,501	0,784	0,085
26	0,33	0,485	0,705	0,066
28	0,33	0,496	0,712	0,044
30	0,173	0,451	0,401	0,027
32	0,161	0,424	0,432	0,018
34	0,207	0,423	0,455	0,018
36	0,152	0,411	0,415	0,013
38	0,102	0,375	0,465	0,012
40	0,201	0,359	0,558	0,011
42	0,155	0,359	0,469	0,011
44	0,181	0,338	0,48	0,009
46	0,267	0,386	0,569	0,009
48	0,167	0,383	0,565	0,01

c

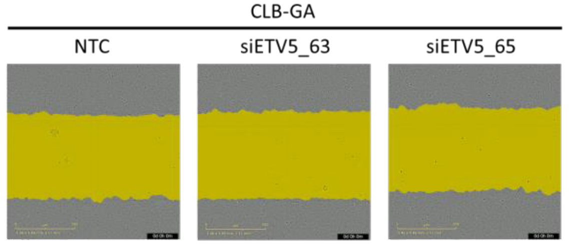

d

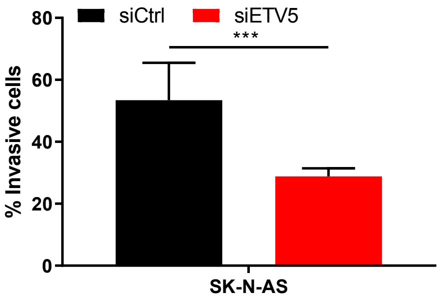

e

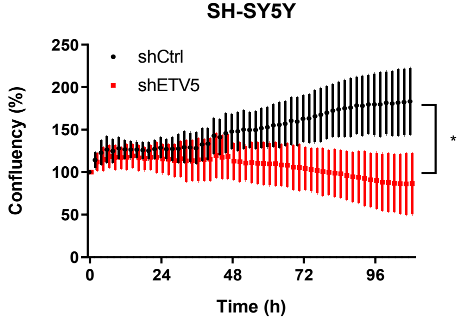

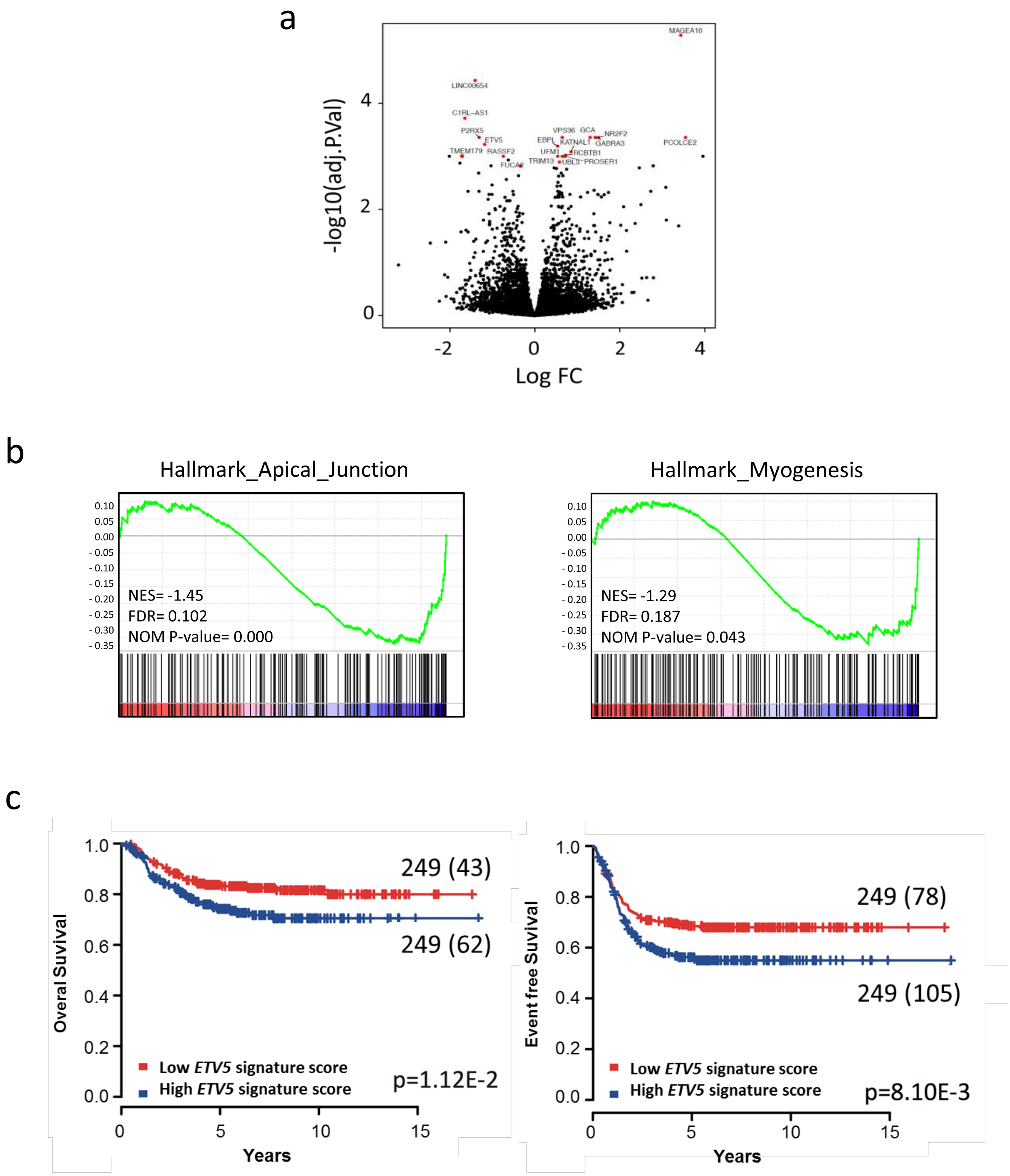
a
MAGEA10
LINC00654
C1RL-AS1
P2RX5
ETV5
TMEM179
RASSF2
FUCA2
VPS36
GCA
NR2F2
EBPL
KATNAL1
GABRA3
PCOLCE2
UFM1
RCBTB1
TRIM13
UBL3
PROSER1
-log10(adj.P.Val)
Log FC
b
Hallmark_Apical_Junction
NES= -1.45
FDR= 0.102
NOM P-value= 0.000
Hallmark_Myogenesis
NES= -1.29
FDR= 0.187
NOM P-value= 0.043
c
249 (43)
249 (62)
Overal Survival
Low ETV5 signature score
High ETV5 signature score
p=1.12E-2
Years
249 (78)
249 (105)
Event free Survival
Low ETV5 signature score
High ETV5 signature score
p=8.10E-3
Years

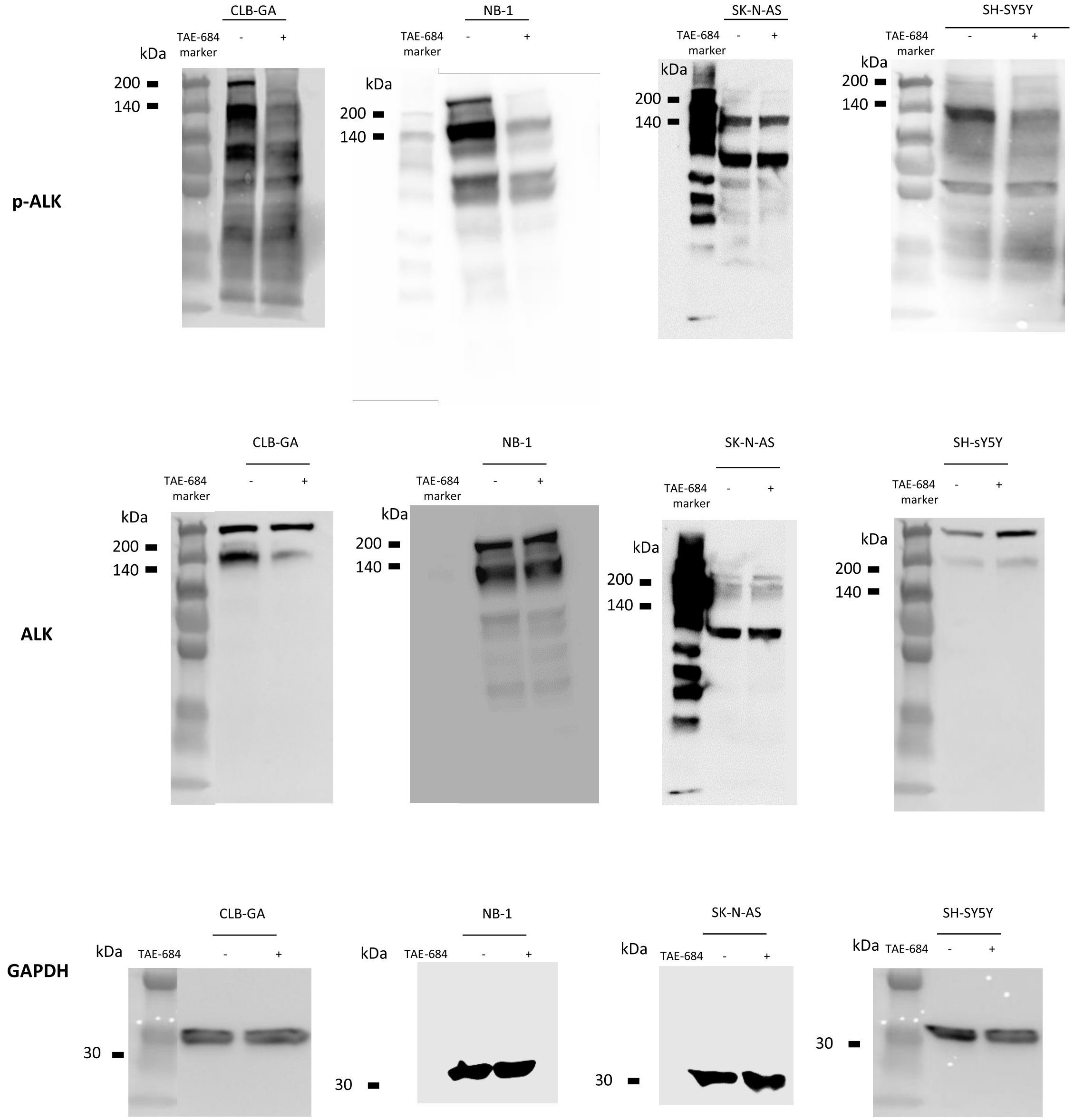

p-ALK
ALK
GAPDH
CLB-GA
NB-1
SK-N-AS
SH-SY5Y
SH-sY5Y
TAE-684
marker
-
+
kDa
200
140
30

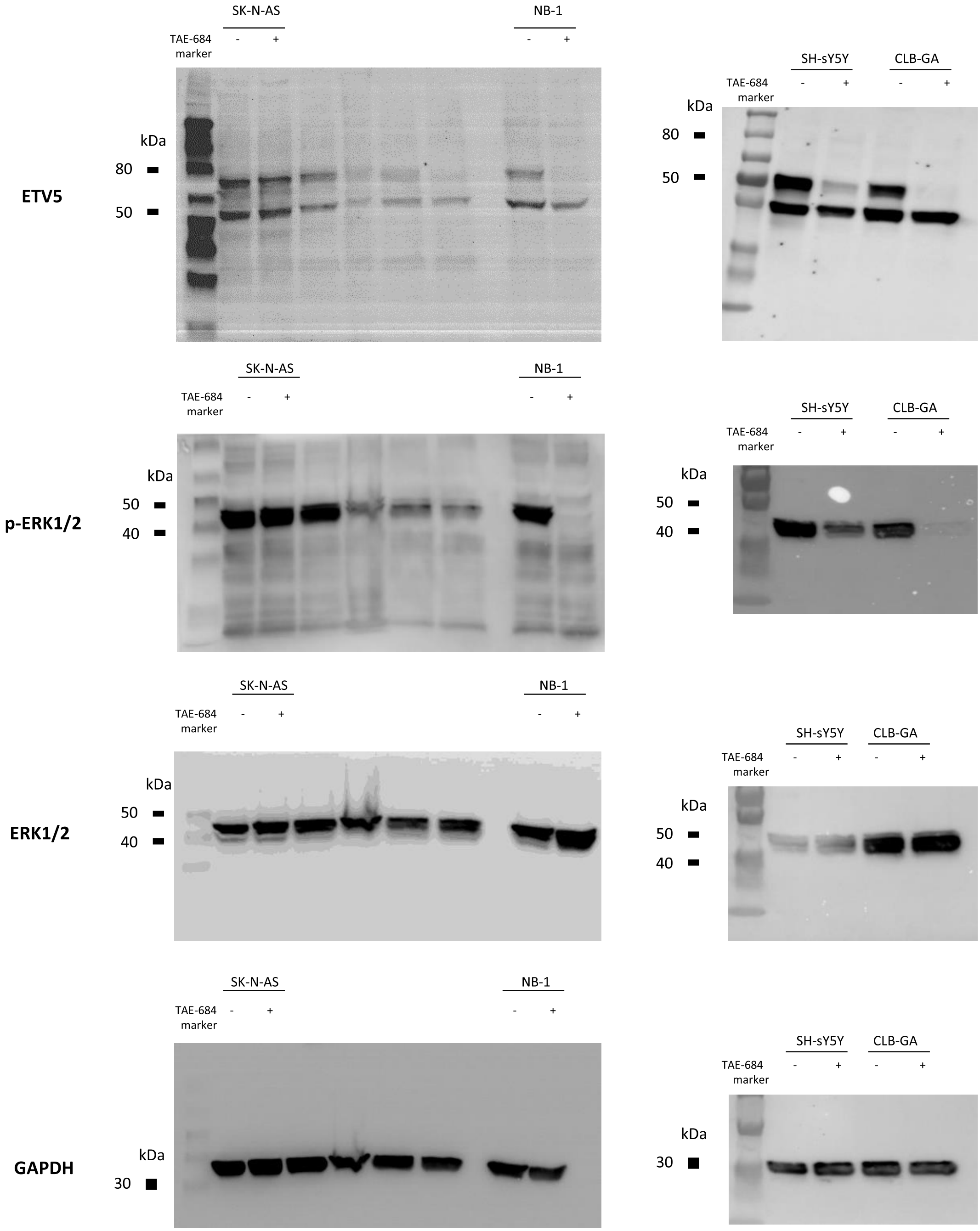

ETV5
p-ERK1/2
ERK1/2
GAPDH
SK-N-AS
NB-1
SH-sY5Y
CLB-GA
TAE-684
marker
- + - +
kDa
80
50
40
30

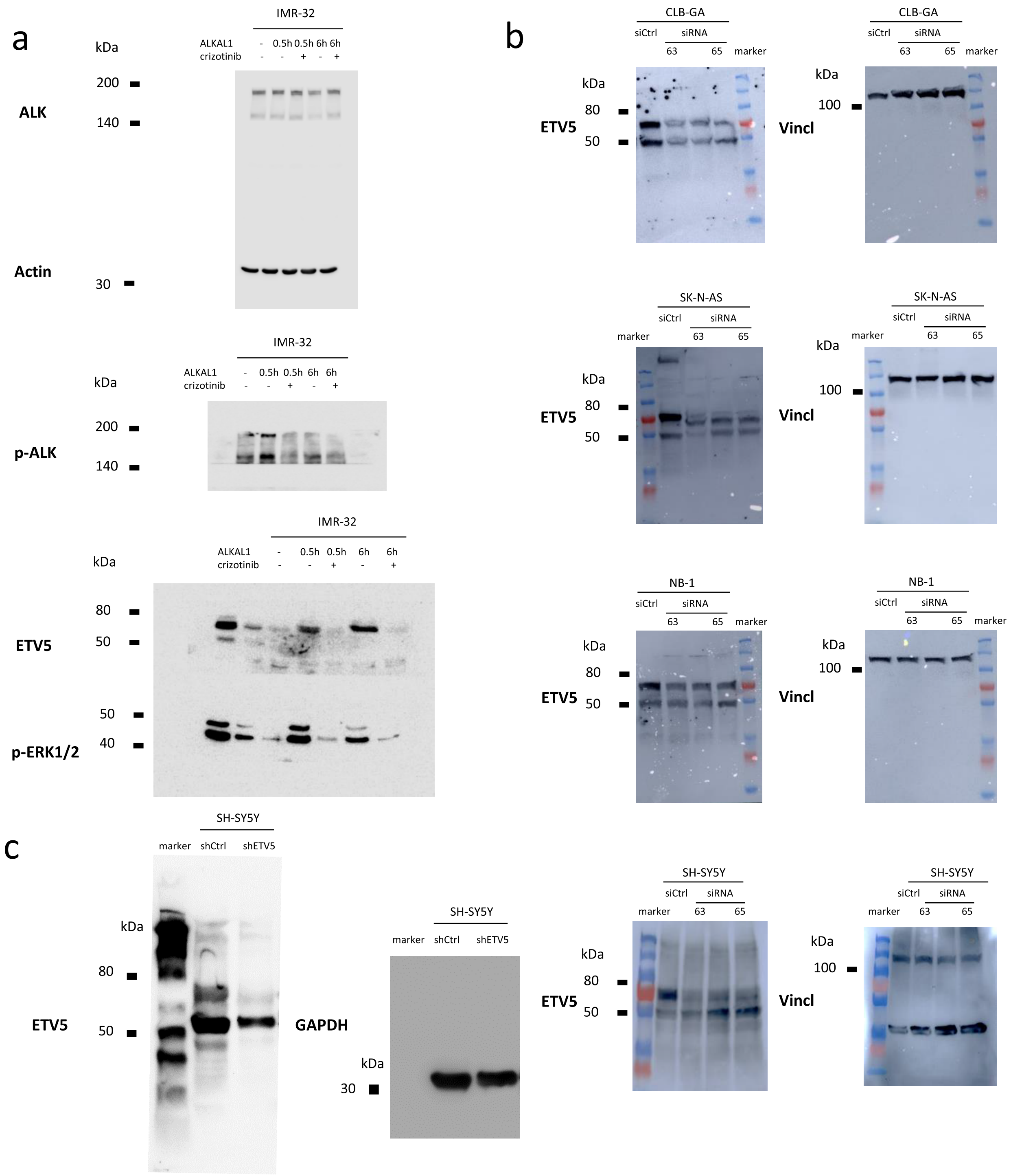

a
IMR-32
kDa
ALKAL1 - 0.5h 0.5h 6h 6h
crizotinib - - + - +
200
140
ALK
Actin
30
IMR-32
kDa
ALKAL1 - 0.5h 0.5h 6h 6h
crizotinib - - + - +
200
140
p-ALK
IMR-32
kDa
ALKAL1 - 0.5h 0.5h 6h 6h
crizotinib - - + - +
80
50
ETV5
50
40
p-ERK1/2
b
CLB-GA
siCtrl siRNA
63 65 marker
kDa
80
50
ETV5
CLB-GA
siCtrl siRNA
63 65 marker
kDa
100
Vincl
SK-N-AS
siCtrl siRNA
marker 63 65
kDa
80
50
ETV5
SK-N-AS
siCtrl siRNA
marker 63 65
kDa
100
Vincl
NB-1
siCtrl siRNA
63 65 marker
kDa
80
50
ETV5
NB-1
siCtrl siRNA
63 65 marker
kDa
100
Vincl
SH-SY5Y
siCtrl siRNA
marker 63 65
kDa
80
50
ETV5
SH-SY5Y
siCtrl siRNA
marker 63 65
kDa
100
Vincl
C
SH-SY5Y
marker shCtrl shETV5
kDa
80
50
ETV5
GAPDH
SH-SY5Y
marker shCtrl shETV5
kDa
30
